# Galectin-1 Attenuates PDGF-Mediated AKT Signaling in Retinal Pigment Epithelial Cells

**DOI:** 10.3390/ijms25179267

**Published:** 2024-08-27

**Authors:** Martina Bizzotto, Annabella Ostermaier, Caspar Liesenhoff, Wenxiu Ma, Arie Geerlof, Siegfried G. Priglinger, Claudia S. Priglinger, Andreas Ohlmann

**Affiliations:** 1 Department of Ophthalmology, University Hospital, LMU Munich, Mathildenstrasse 8, 80336 Munich, Germany; martina.bizzotto@med.uni-muenchen.de (M.B.); annabella.ostermaier@med.uni-muenchen.de (A.O.); caspar.liesenhoff@med.uni-muenchen.de (C.L.); siegfried.priglinger@med.uni-muenchen.de (S.G.P.); claudia.priglinger@med.uni-muenchen.de (C.S.P.); 2 Protein Expression and Purification Facility, Institute of Structural Biology, Helmholtz Center Munich for Environmental Health, 85764 Neuherberg, Germany; arie.geerlof@helmholtz-muenchen.de

**Keywords:** galectin-1, galectin-1 deficiency, AKT signaling, pAKT, ERK signaling, pERK, PDGF, PDGF-Rβ, retinal pigment epithelium cells, ARPE-19

## Abstract

Galectins have the potential to interact with transmembrane glycoproteins to modulate their functions. Since galectin-1 interacts with PDGF-Rβ, we analyzed the effect of galectin-1 on PDGF-BB-mediated AKT signaling in primary human retinal pigment epithelial (RPE) cells and galectin-1-deficient immortalized human RPE cells (LGALS1^−/−^/ARPE-19) following incubation with PDGF-BB and galectin-1. Expression and localization of galectin-1, PDGF-Rβ and pAKT were investigated using western blot analysis and immunohistochemical staining. Cell proliferation of RPE cells was analyzed using BrdU ELISA. Following treatment of human RPE cells with human recombinant (hr)-galectin-1 and PDGF-BB, an intense clustering of PDGF-Rβ and colocalization with galectin-1 were detected. By Western blot analysis and immunocytochemistry of human RPE cells, an enhanced PDGF-BB-mediated expression of pAKT was observed, which was substantially reduced by additional incubation with hr-galectin-1. Vice versa, in LGALS1^−/−^/ARPE-19 cells, the PDGF-BB-induced pAKT signal was enhanced compared to wild-type cells. Furthermore, a decreased expression of PDGF-Rβ in human RPE cells was observed after treatment with PDGF-BB and hr-galectin-1, while in untreated LGALS1^−/−^/ARPE-19 cells, its constitutive expression was increased. In addition, after treatment of RPE cells with hr-galectin-1, the PDGF-BB-induced proliferation was markedly reduced. In summary, galectin-1 has the distinct potential to reduce PDGF-mediated pAKT signaling and proliferation in human RPE cells—an effect that is most likely facilitated via a decreased expression of PDGF-Rβ.

## 1. Introduction

Galectins are endogenous lectins with highly conserved carbohydrate recognition domains that mediate specific binding to β-galactoside moieties [1]. β-galactosylation is a common glycosylation motive of various intra- and extracellular as well as transmembrane proteins and receptors, which facilitates their potential interaction with galectins [2]. In addition to their interaction with β-galactoside-binding sites, galectins can also undergo non-carbohydrate-dependent protein–protein interactions [1,3].

Galectin-1 has one carbohydrate recognition domain (CRD) and forms homodimers via sandwich binding of their two anti-parallel β-sheets, and it is found both intra- and extracellularly [4]. In the cell, galectin-1 was observed in the cytoplasm and the nucleus, where it interacts with several proteins to modulate signaling pathways, transcription, apoptosis, actin polymerization or preRNA processing [4,5,6]. Extracellularly, it is located in the outer plasma membrane, where it binds to glycosylated membrane proteins or receptors, or in the extracellular matrix [4]. Via its two binding sites, galectin-1 homodimers can form an extracellular lattice of membrane proteins or receptors, which in turn can modulate their function, and subsequently, their intracellular signal pathways [5,7]. Due to its various intra- and extracellular functions, galectin-1 is involved in several physiological and pathological processes, including fibrosis, diabetes and obesity, immune response, or tumor development and progression [8,9,10,11].

In the posterior eye segment, the retinal pigment epithelium is essential for the maintenance of the chorioretinal interface and plays a crucial role in various chorioretinal diseases. Intriguingly, a role for galectin-1 as a potential modulator is discussed in several chorioretinal pathologies, such as proliferative vitreoretinopathy (PVR). PVR is a severe complication following retinal detachment, leading to formation of epiretinal and subretinal membranes, and subsequently to recurrent retinal detachment. Intriguingly, within these membranes, which are predominantly formed by transdifferentiated RPE cells, a robust expression for galectin-1 was observed [12]. In the pathogenesis of PVR RPE cell transdifferentiation, proliferation and subsequent membrane formation is driven and maintained by a variety of growth factors, including vascular endothelial growth factor (VEGF), fibroblast growth factor−2, transforming growth factor-β or platelet-derived growth factor [13,14,15].

PDGF is a growth factor that is expressed in a wide range of cells. It consists of four isoforms, which are expressed from four different genes designated as PDGF-A to D [16]. Apart from PDGF-AB, the biologically active forms are homodimers, which all have the potential to bind to PDGF receptor (PDGF-R)α and/or β [17]. PDGF-Rα and β are classical receptor tyrosine kinases, with an extracellular ligand-binding domain and an intracellular tyrosine kinase domain [18]. Following binding, PDGF forms a homo- or heterodimer of its receptors, which in turn induce auto-phosphorylation of their intracellular domain and subsequent activation of different downstream pathways, such as mitogen-activated protein (MAP) kinase, phosphoinositide−3-kinase/protein kinase B (PI3-kinase/Akt) or phospholipase C (PLC)γ pathways, to promote cell biological effects like proliferation or migration [18].

Since retinal pigment epithelium (RPE) cells and PDGF play a critical role in most diseases of the chorioretinal interface, and galectins have the distinct potential to bind to PDGF-Rβ, thereby potentially modifying its intracellular shuttling and signaling [19,20,21], we investigated whether galectin-1 could modulate PDGF signaling in RPE cells. For this purpose, the potential interaction of galectin-1 and PDGF-Rβ was analyzed on isolated human RPE after PDGF-BB treatment via immunohistochemistry. To determine the downstream effects, human RPE cells and immortalized human RPE cells (ARPE-19) with and without a lack of galectin-1 expression (ARPE-19/LGALS1^−/−^) [22] were incubated with PDGF-BB in combination with human recombinant (hr)-galectin-1, and pAKT, pERK1/2 and β-catenin signaling, as well as the glycosylation-dependence of the observed downstream effects, were analyzed. Finally, their effect on cell proliferation was investigated.

## 2. Results

### 2.1. Galectin-1 Colocalizes with PDGF-BB-Induced Clusters of PDGF-Rβ

To investigate whether exogenous galectin-1 interacts with PDGF-Rβ in human RPE cells, cultured human RPE cells were incubated with hr-galectin-1 and/or PDGF-BB and analyzed by immunohistochemistry.

**Figure 1:**
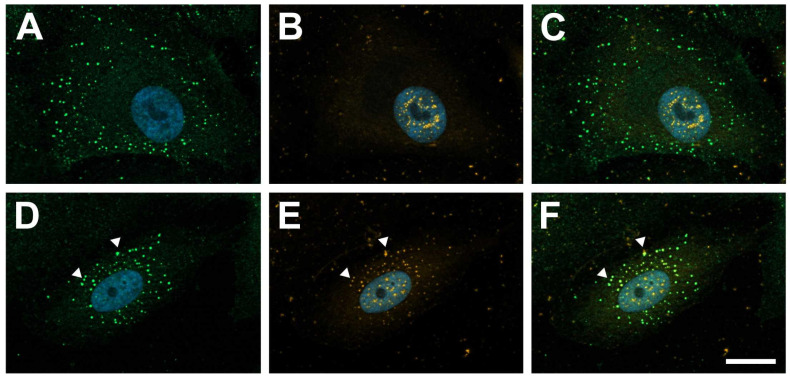
Galectin-1 colocalizes with PDGF-Rβ in PDGF-BB-induced clustering. Immunohistochemical staining for PDGF-Rβ (green) and galectin-1 (orange) of human RPE cells after incubation with 20 ng/mL PDGF-BB (**A**–**C**) or a combination of 20 ng/mL PDGF-BB and 10 µg/mL hr-galectin-1 (**D**–**F**) for 30 min. Arrow heads, colocalization of galectin-1 with PDGF-Rβ; blue, DAPI staining; scale bar, 20 µm.

Following treatment of human RPE cells with PGDF-BB for 30 min, a punctate staining for PDGF-Rβ was observed on the cell membrane, suggesting a PDGF-BB-induced clustering of this receptor (Figure 1A,C), whereas staining for endogenous galectin-1 was predominantly located in the nucleus (Figure 1B,C), as it was in untreated controls. No obvious colocalization between PDGF-Rβ and endogenous galectin-1 was observed following treatment with PDGF-BB alone. After incubation of human cultured RPE cells with hr-galectin-1 and PDGF-BB, the punctate staining for PDGF-Rβ was detected at the plasma membrane again, although it showed a more perinuclear localization (Figure 1D,F). In addition, within the PDGF-BB-mediated clusters of PDGF-Rβ, an intense signal for galectin-1 was observed (arrow heads in Figure 1D,F), strongly suggesting an interaction of galectin-1 with PDGF-Rβ in cultured human RPE cells.

### 2.2. PDGF-BB-Induced AKT and ERK1/2 Signaling Is Reduced by hr-Galectin-1

Next, we investigated whether the interaction of galectin-1 with PDGF-Rβ modifies the intracellular PDGF-BB-mediated signaling. To achieve this, the expression levels for pAKT, pERK1/2 and β-catenin were analyzed on human cultured RPE cells via Western blot analyses and immunohistochemical staining after treatment with PDGF-BB, hr-galectin-1 or a combination thereof.

**Figure 2:**
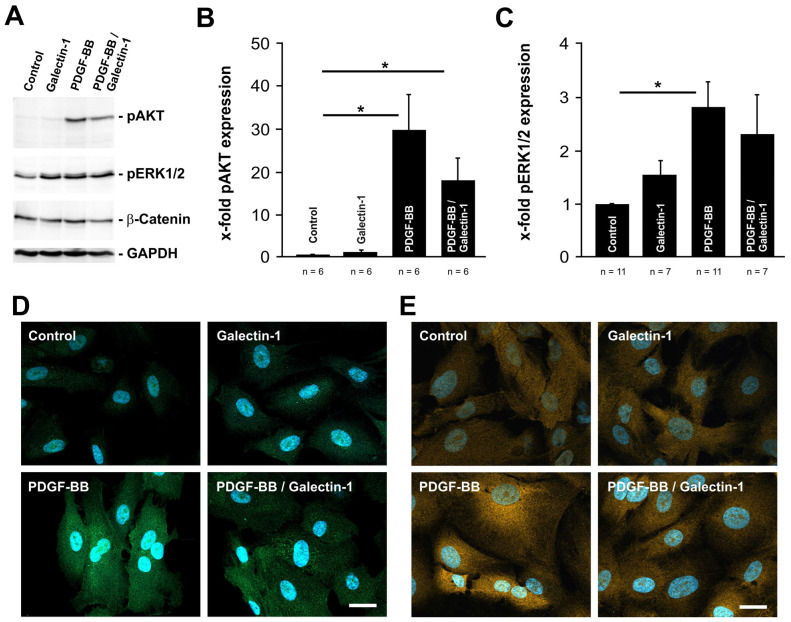
PDGF-BB-induced AKT and ERK1/2 signaling is reduced by hr-galectin-1. (**A**–**C**). Western blot analysis (**A**) and densitometry (**B**,**C**) for pAKT, pERK1/2 and β-catenin of human RPE cells after treatment with 10 µg/mL hr-galectin-1 and/or 20 ng/mL PDGF-BB for 30 min. Mean ± SEM; * *p* < 0.05. (**D**,**E**) Representative immunohistochemical staining against pAKT (**D**) and pERK1/2 (**E**) of human RPE cells after incubation with 10 µg/mL hr-galectin-1 and/or 20 ng/mL PDGF-BB for 30 min. Blue, DAPI staining; scale bar, 50 µm.

In untreated control cells, a weak but specific band for pAKT was observed, which was similar to that of hr-galectin-1-treated cells (Figure 2A,B). In contrast, following incubation with PDGF-BB for 30 min, a strong signal for pAKT was detected, which was 30.0 ± 8.7-fold higher compared to untreated controls (Figure 2A,B). Intriguingly, the combined treatment with hr-galectin-1 and PDGF-BB reduced the PDGF-BB-mediated increase in pAKT to 18.1 ± 5.2-fold compared to untreated controls (Figure 2A,B). Thus, the addition of hr-galectin-1 decreased the PDGF-mediated enhanced phosphorylation of AKT to 60.5 ± 17.4%—a difference that was statistically significant (100 ± 29.0%; *n* = 8; *p* = 0.033). 

Homologous results, although to a lesser extent, were obtained for pERK1/2 signaling. Again, following the incubation of human RPE cells with PDGF-BB, a significant increase in pERK expression of 2.8 ± 0.4-fold was detected compared to untreated controls (Figure 2A,C). However, the additive treatment with hr-galectin-1 only led to a slight decline in PDGF-BB-mediated ERK phosphorylation (2.3 ± 0.7-fold) by approximately 18% (Figure 2A,C). Cultured human RPE cells that were only incubated with hr-galectin-1 showed a weak increase in ERK1/2 phosphorylation (1.55 ± 0.25-fold). 

Furthermore, only minor effects on the expression of β-catenin in RPE cells were observed following PDGF-BB and/or hr-galectin-1 treatment (Figure 2A). 

In line with this, immunohistochemical analysis revealed a marked increase in pAKT and pERK labeling in PDGF-BB incubated human cultured RPE cells when compared to untreated controls and hr-galectin-1 incubated cells (Figure 2D,E). Again, following the treatment with PDGF-BB and hr-galectin-1, the signal for pAKT and pERK was considerably lower compared to PDGF-BB incubated RPE cells (Figure 2D,E). Overall, our data strongly suggest that galectin-1 can reduce pAKT and, to a lesser extent, pERK signaling. 

### 2.3. Galectin-1 Reduces PDGF-BB-Induced AKT Signaling in a Glycosylation-Dependent Manner

Influence of β1,6-N-glycosylation on PDGF-BB-mediated upregulation of pAKT and its modulation by hr-galectin-1.

**Figure 3:**
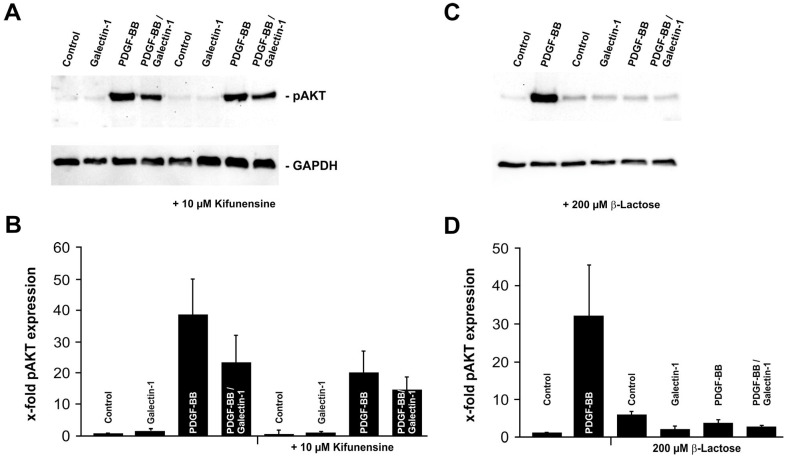
Galectin-1 reduces PDGF-BB-induced AKT signaling in a glycosylation-dependent manner. Western blot analysis (**A**,**C**) and densitometry (**B**,**D**) for pAKT of human RPE cells after pretreatment with 10 µM kifunensine for 4 d (**A**,**B**) or β-lactose immediately before the experiment (**C**,**D**) and incubation with 10 µg/mL hr-galectin-1 and/or 20 ng/mL PDGF-BB for 30 min. Mean ± SEM; *n* = 6 for (**B**), *n* = 3 for (**D**).

As described before, galectin-1 is a carbohydrate-binding protein that mediates its function via galectin–carbohydrate but also galectin–protein interactions. To analyze whether hr-galectin-1 modulated pAKT signaling is glycosylation-dependent, human RPE cells were pretreated with kifunensine for 4 days. Kifunensine is a selective inhibitor of Golgi class I α-mannosidases [23,24,25], which prevents complex-type *β1,6-N*-glycosylation of proteins and thereby reduces the binding of galectin-1 to its binding partners [21]. As observed before, pAKT signaling was substantially increased in human RPE cells following the treatment with PDGF-BB (38.9 ± 11.2-fold) compared to untreated controls—an effect that was reduced to approximately 60% when cells were additionally incubated with hr-galectin-1 (23.4 ± 8.8-fold; Figure 3A,B). When kifunesine-treated cells were incubated with PDGF-BB alone, the signal for pAKT was enhanced (20.0 ± 7.1-fold), but the increase was only 51.5 ± 18.2% of that of PDGF-BB incubated RPE cells without kifunensine pretreatment (100 ± 28.8%, *p* = 0.022; Figure 3A,B).

In line with this, the combined incubation of kifunensine-pretreated RPE cells with hr-galectin-1 and PDGF-BB also led to an increase in pAKT signaling, but again, this rise was not as pronounced as in cells without kifunensine treatment (14.5 ± 4.2-fold with kifunensine vs. 23.4 ± 8.8-fold without kifunensine; Figure 3A,B). Furthermore, the reduced *β1,6*-*N*-glycosylation lowered the impact of hr-galectin-1 on mitigating PDGF-BB-induced upregulation of pAKT, which was not as strong as in cells without prior kifunensine treatment (reduction in pAKT levels by 25.2% with kifunesine pretreatment vs. 38.7% without kifunensine pretreatment; Figure 3A,B). Thus, *β1,6*-*N*-glycosylation in human RPE cells is required for both for PDGF-BB-induced activation of the AKT pathway and for galectin-1-mediated modulation of pAKT signaling. 

Inhibition of β-galactoside interactions reduces PDGF-BB- and hr-galectin-1-mediated AKT activation.

In order to further confirm the relevance of carbohydrate binding in galectin-1- and PDGF-BB-mediated pAKT modulation in RPE cells, β-lactose was used as a competitive inhibitor for β-galactoside-dependent interactions. β-lactose can block the β-galactoside-dependent protein interaction via binding to the carbohydrate recognition domain of galectins. Therefore, this assay is routinely used to demonstrate the carbohydrate dependence of galectin functions as opposed to protein–protein interactions, which would not be influenced by addition of β-lactose [12,26].

As described before, following the treatment of human RPE cells with PDGF-BB, a robust band for pAKT was detected compared to controls (Figure 3C,D). However, after the incubation of β-lactose-treated human RPE cells with PDGF-BB and/or hr-galectin-1, no enhanced signal for pAKT was detected, neither after the addition of PDGF-BB nor with PDGF-BB and hr-galectin-1 (Figure 3C,D), confirming that both PDGF-BB itself and hr-galectin-1 require β-galactoside moieties to mediate AKT signaling. 

### 2.4. Lack of Endogenous Galectin-1 Enhances PDGF-BB-Mediated pAKT Signaling

Since we could demonstrate that exogenously added hr-galectin-1 reduces PDGF-BB-mediated pAKT signaling in a glycosylation-dependent manner, we wanted to ascertain whether lack of endogenous galectin-1 leads to its enhancement. To this end, we used an immortalized RPE cell line (ARPE-19) with a CRISPR/Cas9-mediated knockout of the galectin-1 gene (ARPE-19/LGALS1^−/−^), which has been characterized previously [22].

**Figure 4:**
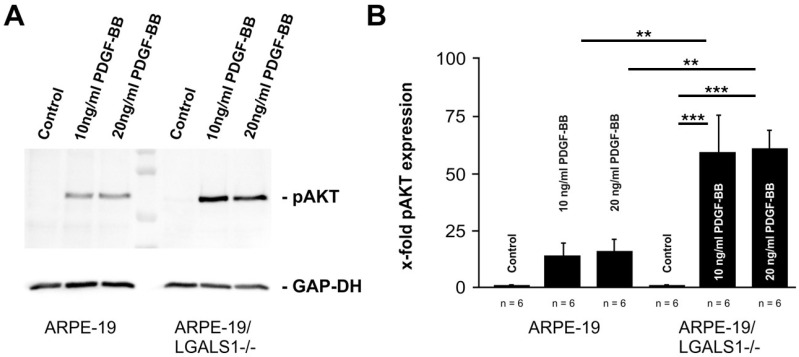
Lack of galectin-1 enhances PDGF-BB-mediated pAKT signaling. Western blot analysis (**A**) and densitometry (**B**) of galectin-1-deficient immortalized RPE cells (ARPE-19/LGALS1^−/−^) and wild-type controls (ARPE-19) following treatment with 10 or 20 ng/mL PDGF-BB for 30 min. Mean ± SEM; ** *p* < 0.01, *** *p* < 0.001.

Following treatment of wild-type ARPE-19 cells with 10 and 20 ng/mL PDGF-BB, an increased signal for pAKT was observed, which was 14.3 ± 7.2- and 15.5 ± 7.8-fold, respectively, when compared to untreated controls (Figure 4A,B). In galectin-1-deficient ARPE-19 cells, however, the increase in pAKT following PDGF-BB treatment with 10 and 20 ng/mL was even more marked, with a 58.9 ± 16.6- and 60.8 ± 8.9-fold enhancement, respectively, compared to untreated ARPE-19/LGALS1^−/−^ cells (*n* = 6; *p* = 0.0003 for 10 and 20 ng/mL; Figure 4A,B). Compared with PDGF-BB-treated wild-type controls, the pAKT signal was approximately 4 times higher in ARPE-19/LGALS1^−/−^ cells, which was a statistically significant difference (*p* = 0.0059 for 10 ng/mL; *p* = 0.0083 for 20 ng/mL; Figure 4A,B). Overall, our data strongly suggest that the lack of galectin-1 leads to enhanced pAKT signaling following PDGF-BB treatment.

### 2.5. Galectin-1 Reduces PDGF-BB-Mediated Clustering of PDGF-Rβ 

Since we could demonstrate that galectin-1 reduces PDGF-BB-mediated AKT signaling, we investigated whether galectin-1 mediates its effects via modulation of PDGF-BB-induced clustering of PDGF-Rβ in cultured human RPE cells by immunohistochemistry.

**Figure 5:**
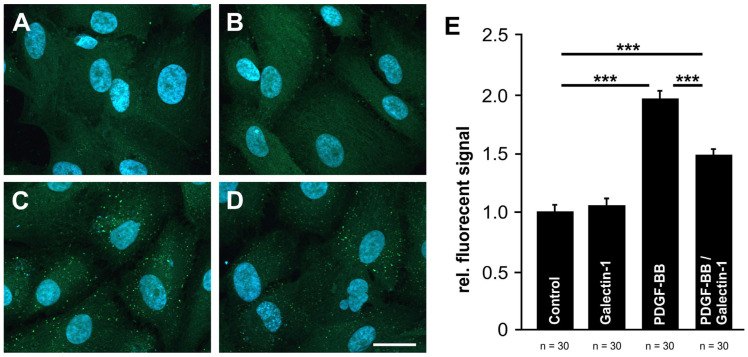
Galectin-1 reduces PDGF-BB-mediated clustering of PDGF-Rβ. (**A**–**D**). Immunohistochemical staining for PDGF-Rβ (green) of untreated human RPE cells (**A**) and after incubation with 20 ng/mL PDGF-BB (**B**), 10 µg/mL hr-galectin-1 (**C**) and a combination of 20 ng/mL PDGF-BB and 10 µg/mL hr-galectin-1 (**D**) for 30 min. Blue, DAPI staining; scale bar, 30 µm. (**E**). For quantification, the fluorescent signal was analyzed and plotted as relative fluorescent signal in relation to the control group. Mean ± SEM of three independent experiments; *** *p* < 0.001.

In untreated human RPE cells, as well as following treatment with rh-galectin-1, only a weak and rather diffuse signal for PDGF-Rβ could be detected (Figure 5A,B). As shown before, the treatment with PDGF-BB induced an enhanced, spotted fluorescent labeling for PDGF-Rβ at the cell surface (Figure 5C), indicating clustering of PDGF-Rβ. Intriguingly, after additional incubation of RPE cells with hr-galectin-1, the PDGF-BB-mediated clustering was markedly reduced when compared to cells treated with PDGF-BB alone (Figure 5D).

To further confirm our observations, the fluorescent signal for PDGF-Rβ was quantified. The treatment of human RPE cells with hr-galectin-1 had only minor effects on the fluorescent signal (1.06 ± 0.04-fold) compared to untreated controls (1.0 ± 0.05-fold; Figure 5E). However, following incubation of the cells with PDGF-BB, the fluorescent signal for PDGF-Rβ was significantly increased to 1.95 ± 0.06-fold—an effect that was substantially blocked when hr-galectin-1 was additionally added (1.48 ± 0.04-fold). Overall, our data strongly suggest that galectin-1 can attenuate PDGF-BB-mediated clustering.

### 2.6. Exogenous and Endogenous Galectin-1 Mitigates PDGF-BB-Mediated pAKT Signaling by Reducing PDGF-Rβ Expression in RPE Cells

To provide further insight on the mechanism of galectin-1-mediated attenuation of PDGF-BB-induced AKT signaling and reduced PDGF-Rβ clustering, the protein expression levels for PDGF-Rβ were determined using Western blot analyses. 

**Figure 6:**
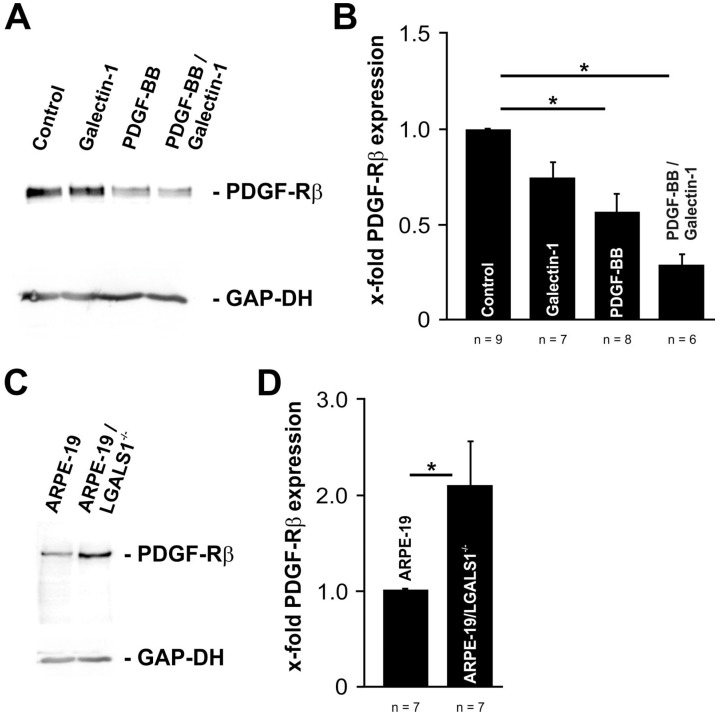
Galectin-1 reduces PDGF-Rβ expression in RPE cells. A,B. Western blot analysis (**A**) and densitometry (**B**) of cultured RPE cells for PDGF-Rβ following treatment with 10 µg/mL hr-galectin-1 and/or 20 ng/mL PDGF-BB for 30 min. Mean ± SEM; * *p* < 0.05. (**C**,**D**) Western blot analysis (**C**) and densitometry (**D**) of galectin-1-deficient immortalized RPE cells (ARPE-19/LGALS1^−/−^) and wild-type controls (ARPE-19). Mean ± SEM; * *p* < 0.05.

In untreated RPE cells, a prominent band for PDGF-Rβ was detected, which was less intense when cells were treated with hr-galectin-1 (72.9 ± 8.5%) or PDGF-BB (56.1 ± 9.0%, *p* = 0.014; Figure 6A,B). Intriguingly, following the treatment of RPE cells with hr-galectin-1 and PDGF-BB, the signal for PDGF-Rβ was even lower than for single treatments (28.5 ± 5.5%, *p* = 0.011; Figure 6A,B). When compared directly, the signal for PDGF-Rβ was 49% ± 9.7% lower in double-treated than in PDGF-BB-incubated RPE cells (100 ± 16.0%)—a difference that was statistically significant (*p* = 0.024).

Vice versa, in immortalized galectin-1-deficient ARPE-19 cellsthe protein expression levels for PDGF-Rβ were significantly higher (2.1- ± 0.5-fold, *p* < 0.032) compared to wild-type controls (Figure 6C,D). Overall, our data strongly suggest that both exogenously added and endogenously expressed galectin-1 has the distinct potential to downregulate the protein expression of PDGF-Rβ. 

### 2.7. Galectin-1 Attenuates PDGF-Induced RPE Cell Proliferation

To investigate whether the interaction of PDGF-Rβ and galectin-1 and its influence on PDGF-BB-induced pAKT signaling result in a downstream functional effect, cells were treated with PDGF-BB with or without the addition of hr-galectin-1, and the proliferation of cultured human RPE cells was assessed by BrdU ELISA.

**Figure 7:**
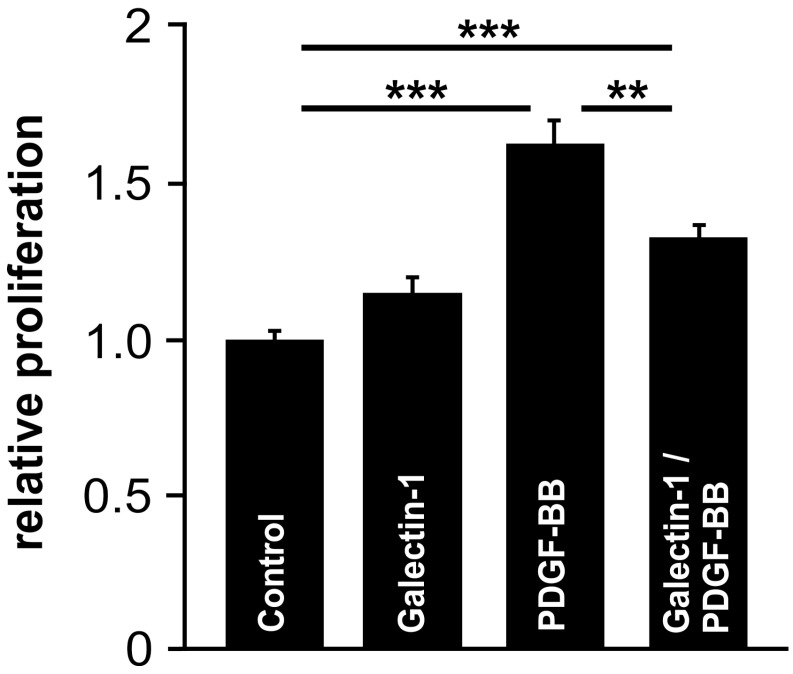
Galectin-1 attenuates PDGF-BB-induced RPE cell proliferation. BrdU ELISA assessment of RPE cells following incubation with 10 µg/mL hr-galectin-1 and/or 20 ng/mL PDGF for 24 h. Mean ± SEM; *n* = 20 of five independent experiments; ** *p* < 0.01, *** *p* < 0.001.

Following incubation of cells with hr-galectin-1 alone, a slight increase in cell proliferation was observed, while the treatment of RPE cells with PDGF-BB for 24 h led to an increased proliferation of 62.7 ± 7.4% (*p* < 0.001) compared to untreated controls (Figure 7). However, the PDGF-BB-mediated proliferative effect was reduced to 32.2 ± 4.6% when cells were additionally incubated with hr-galectin-1—an effect that was statistically significant (*p* < 0.01; Figure 7). Overall, our data strongly suggest that galectin-1-induced inhibition of PDGF-BB signaling leads to cell biological effects, such as reduced proliferation of RPE cells.

## 3. Discussion

In our present study, we showed that both hr-galectin-1 and endogenous galectin-1 have the potential to attenuate PDGF-BB-mediated pAKT signaling in human RPE cells via a reduced expression of PDGF-Rβ. Our conclusions rest upon (1) the observation that the interaction of PDGF-Rβ with hr-galectin-1 leads to decreased pAKT signaling; (2) the finding that in galectin-1-deficient immortalized RPE cells, the pAKT signal was significantly enhanced following PDGF-BB treatment; (3) the potential of rh-galectin-1 to reduce PDGF-BB-induced receptor clustering and expression of PDGF-Rβ; (4) the observation of an enhanced expression of PDGF-Rβ in galectin-1-deficient RPE; and finally, (5) the ability of hr-galectin-1 to reduce PDGF-BB-induced proliferation of RPE cells. 

Galectin-1 is a protein that interacts with several intra- and extracellular proteins to modulate apoptotic processes, preRNA processing, transcription or signaling pathways [4,5,6,7]. In previous works, a glycosylation-dependent interaction between PDGF-Rβ and galectin−3 has been reported, which in turn promoted the clustering of both proteins and a moderate phosphorylation of ERK1/2, AKT and GSK−3α/β [21]. In line with this, by employing immunohistochemical analysis, we observed a colocalization of PDGF-Rβ with galectin-1 in RPE cells following the treatment with PDGF-BB and hr-galectin-1, strongly suggesting an interaction of both proteins at the cell surface. 

PDGF-Rβ belongs to class III receptor tyrosine kinases that activate several intracellular pathways, including MAP kinase, AKT, PLCγ, β-catenin or signal transducer and activator of transcription (STAT) following the binding of PDGF-BB [27,28,29]. After treatment of human RPE cells with PDGF-BB, we observed a significant increase in phosphorylated AKT and ERK1/2, while the expression of β-catenin remained unchanged, indicating a selective effect of PDGF-BB on these signaling pathways in RPE cells, which is in agreement with previous observations [30]. Intriguingly, the additional incubation of cells with hr-galectin-1 led to a reduction in PDGF-BB-induced pAKT and pERK levels. Vice versa, in immortalized RPE cells with targeted deletion of the galectin-1 gene, the pAKT signal was significantly enhanced following PDGF-BB treatment, strongly suggesting that galectin-1 can modulate the intensity of this pathway, irrespective of whether it is added exogenously or endogenously expressed. A comparable influence of galectin-1 was found in PDGF-BB-treated airway smooth muscle cells, where galectin-1 reduced pAKT expression, which in turn led to reduced migration and proliferation [20]. Nevertheless, the influence of galectin-1 on AKT and ERK signaling appears to vary and depend on several factors, including cell type, treatment, glycosylation, pathological conditions and other biological contexts [31,32,33]. For instance, in myeloma cell lines, but not in B-cell lines, galectin-1 promotes survival via an enhanced signaling of pAKT and pERK1/2—an effect that depends on the expression of a specific isoform of CD45 [31]. Furthermore, we found that the potential of recombinant galectin-1 to reduce PDGF-BB-mediated pAKT signaling is most likely mediated by a carbohydrate-dependent binding to PDGF-Rβ. 

Pretreatment with two inhibitors of the β-galactoside-dependent carbohydrate–protein interaction markedly reduced PDGF-BB-mediated pAKT signaling in RPE cells. This finding provides evidence that the state of glycosylation of PDGF-Rβ itself can modulate PDGF-BB-induced AKT signaling, which is in line with a previous report on tenon-derived fibroblasts [34]. In addition, both kifunensine and β-lactose substantially mitigated the capacity of hr-galectin-1 to attenuate PDGF-BB-induced AKT phosphorylation in RPE cells, strongly supporting our hypothesis of a specific glycosylation-dependent effect of galectin-1 on the downstream action of PDGF-Rβ. 

Following internalization via clathrin-mediated or clathrin-independent endocytosis, the receptor tyrosine kinases are shuttled to early endosomes from where they are either transferred back to the plasma membrane or tagged for lysosomal degradation [35]. For PDGF-Rβ, both endocytic pathways were detected, leading to cell-type-specific biological effects after stimulation with PDGF-BB [19]. Intriguingly, galectin−3 has been found to be involved in clathrin-independent endocytosis of PDGF-Rβ in fibroblasts, although it is unclear whether it affects intracellular shuttling of the endosomes [19]. Furthermore, galectin−3 and −9 can accumulate in the lysosomal compartment of shigella infected Hela and prostate cancer cells and have been associated with ubiquitinated proteins [36]. Following the incubation of RPE cells with PDGF-BB together with galectin-1, we observed a reduced clustering and lower protein expression of PDGF-Rβ compared to the treatment with PDGF-BB alone. Vice versa, in immortalized ARPE−19/LGALS1^−/−^ cells, the expression level of PDGF-Rβ was increased. Although not investigated in this study, it is tempting to speculate whether galectin-1 may promote enhanced lysosomal degradation of PDGF-Rβ following PDGF-BB stimulation in human RPE cells—an effect of galectins, which has been observed before for other glycoproteins and cell types [37,38].

Proliferative vitreoretinopathy (PVR) is a devastating vision-threatening complication following retinal detachment that is characterized by the formation of cell-rich, contractile epiretinal membranes composed of various transdifferentiated retinal cell types in a proliferative state [39]. Proliferation, migration and attachment of detached RPE cells are key cellular events in the pathogenesis of PVR. Intriguingly, galectin-1 is upregulated in transdifferentiated RPE cells and expressed in PVR membranes [12]. Furthermore, immunohistochemical studies evidenced an expression of PDGF-Rβ and PDGF-BB in these membranes, together with several other growth factors and their receptors [12,40]. The evidence for a pathogenic relevance of PDGF-BB in PVR is derived from our own studies on RPE cells from epiretinal membranes. In these cells, PDGF-BB activated AKT signaling, while depletion of PDGF-Rβ led to attenuated signaling and reduced proliferation, migration and contraction of these cells following incubation with the vitreous of patients suffering from PVR [41]. Since we observed an inhibition of attachment, spreading and migration of transdifferentiated RPE cells by hr-galectin-1 in a previous work [42], and we now demonstrated that galectin-1 attenuates PDGF-BB-mediated proliferation of RPE cells via attenuation of PDGF-BB-induced AKT signaling, it is tempting to speculate that in vitreo-retinal diseases involving transdifferentiated RPE cells, galectin-1 may function to block an exaggerated wound-healing response, as observed in severe PVR.

Overall, we conclude that galectin-1 has the distinct potential to reduce PDGF-mediated pAKT signaling and proliferation in retinal pigment epithelial cells in a glycosylation-dependent manner—an effect that is most likely facilitated by a decreased expression of PDGF-Rβ.

## 4. Methods

### 4.1. Cell Culture

Cultured human RPE cells were isolated from the eyes of human postmortem donors, as described previously [42,43]. The methods for securing human tissue were humane, included proper consent and approval, complied with the Declaration of Helsinki and were approved by the local ethics committee (Ethics Committee of the LMU Munich, Project-Nr. 19–124).

In brief, following the removal of the anterior segment, the neural retina was carefully peeled from the RPE. The eyecup was rinsed with Ca^2 +^ and Mg^2+^-free Hanks’ balanced salt solution and incubated with 0.25% trypsin (Thermo Fisher Scientific, Waltham, MA, USA) for 30 min at 37 °C. After replacement of the trypsin solution with Dulbecco’s modified Eagle’s medium (DMEM; Bio&Sell, Nuernberg, Germany) supplemented with 20% fetal calf serum (Thermo Fisher Scientific, Waltham, MA, USA), cell culture medium was gently agitated to release RPE cells from Bruch’s membrane by avoiding its damage. The harvested RPE cells were transferred into a 6-well cell culture dish and checked for cross-contamination via light microscopy. Following isolation, human RPE cells were cultured in DMEM cell culture medium supplemented with 20% FCS, 50 μg/mL penicillin and 50 μg/mL streptomycin (Thermo Fisher Scientific, Waltham, MA, USA). For subculturing, primary human RPE cells were maintained in DMEM supplemented with 10% FCS, 50 μg/mL penicillin and 50 μg/mL streptomycin (Thermo Fisher Scientific, Waltham, MA, USA), which was changed every second day. The epithelial origin of RPE cells was confirmed by immunostaining against cytokeratin−8 antibodies (Merck, Darmstadt, Germany). 

ARPE−19 cells (ATCC, Manassas, VA, USA) were cultured in DMEM/HAM’s F12 cell culture medium (Bio&Sell, Nuernberg, Germany) supplemented with 10% fetal calf serum, 50 μg/mL penicillin and 50 μg/mL streptomycin (Thermo Fisher Scientific, Waltham, MA, USA), which was changed every other day. 

Human cultured RPE and ARPE−19 cells were maintained under standard culture conditions in an incubator (Thermo Fisher Scientific, Waltham, MA, USA) at 37 °C and 5% CO_2_. Both cell lines were split at a confluence of 75% using versene solution with 0.05% trypsin (both from Thermo Fisher Scientific, Waltham, MA, USA). Unless otherwise indicated, cells were starved in culture medium without supplements for 24 h before the experiments. For immunocytochemistry, ARPE−19 cells were seeded on glass coverslips. For blocking experiments, human RPE cells were incubated with 10 µM kifunensine (Merck, Darmstadt, Germany) for 4 d or with 200 mM β-lactose (Merck, Darmstadt, Germany) immediately before the experiment.

### 4.2. Isolation of hr-Galectin-1 

Human recombinant (hr)-galectin-1 was isolated, as described previously [44,45]. In brief, pETM−11/hgalectin1 transformed *E. coli* cells (strain BL21(DE3); New England Biolabs, Ipswich, MA, USA) were cultured at 20 °C in 500 mL ZYM 5052 auto-induction medium and 100 μg/mL kanamycin (Merck, Darmstadt, Germany). After reaching saturation, cells were harvested via centrifugation, resuspended in 30 mL lysis buffer (20 mM Tris-HCl, 150 mM NaCl, 10 mM MgSO4, 10 μg/mL DNase1, 1 mM AEBSF.HCl, 0.03% (*v*/*v*) CHAPS, 1 mg/mL lysozyme, pH 7.5) and lysed via sonication. Following the centrifugation (40,000× *g*) and filtration (0.2 μm) of the lysates, supernatants were applied onto 2 mL lactose-agarose columns (J-Oil Mills, Tokyo, Japan), which were equilibrated with buffer A (20 mM Tris-HCl, 150 mM NaCl, 0.03% (*v*/*v*) CHAPS, pH 7.5). After washing with 25 mL buffer A three times, the bound proteins were eluted two times with 5 mL buffer A containing 0.2 M β-lactose. Following dialysis against 1× PBS overnight at 4 °C, the dialysate was filtered (0.2 μm) and stored at 4 °C. Protein concentration was determined by measuring the absorbance at 280 nm.

### 4.3. Cell Proliferation

Cell proliferation of human RPE cells was analyzed using the 5-bromo−2′-deoxyuridine (BrdU) ELISA (Merck, Darmstadt, Germany), in accordance with the manufacturer’s recommendations. In brief, 4000 cells per well were seeded into a 96-well plate and incubated for 24 h under standard conditions to ensure complete cell adherence. Following an additional incubation with 20 ng/mL human recombinant PDGF-BB (Bio-Techne, Minneapolis, MN, USA) and/or 10 µg/mL hr-galectin-1 in cell culture medium without supplements containing BrdU labeling solution for 24 h, cells were fixed and incubated with anti-BrdU antibodies. After colorimetric development, the amount of BrdU incorporation into the DNA was quantified by absorbance measurement at a wavelength of 450 nm and a reference at 690 nm on the SpectraMax 190 ELISA reader (Molecular Devices, San Jose, CA, USA).

### 4.4. Immunocytochemistry

After fixation with 4% paraformaldehyde for 10 min, cells were washed 3 × 5 min with 0.1 M phosphate buffer (0.1 M Na_2_HPO_4_ × 2H_2_O, 0.1 M NaH_2_PO_4_ × H_2_O, pH 7.4) and blocked with 3% bovine serum albumin, 0.1% Triton X−100 in 0.1 M phosphate buffer for 30 min. Following incubation with 1:100 rabbit α-galectin-1 (Abcam, Cambridge, UK), 1:100 rabbit α-phospho-Akt (Ser473, Cell Signaling Technology, Cambridge, UK), 1:100 mouse α-PDGF-Rβ (Bio-Techne, Minneapolis, MN, USA) and 1:100 rabbit α-phospho-ERK1/2 (Thr202/Tyr204, Cell Signaling Technology, Cambridge, UK) in 0.3% bovine serum albumin, 0.01% Triton X−100 in 0.1 M phosphate buffer at 4 °C overnight, specimens were washed 3 times for 10 min each with 0.1 M phosphate buffer and incubated with 1:1000 goat anti-rabbit Alexa Fluor 488 or goat anti-mouse Alexa Fluor 555 (both from Thermo Fisher Scientific, Waltham, MA, USA) in 1:10 diluted blocking solution for 1 h at room temperature. After nuclear staining with Hoechst 33342 (Thermo Fisher Scientific, Waltham, MA, USA), specimens were washed again 3 times with 0.1 M phosphate buffer and mounted with the ProLong glass antifade mounting medium (Thermo Fisher Scientific, Waltham, MA, USA). Immunofluorescence staining was analyzed using an Axio Observer 7 fluorescence microscope with an Apotome 2 module (Zeiss, Oberkochen, Germany) and documented using the ZEN software Blue edition 3.0 (Zeiss, Oberkochen, Germany). For quantification of PDGF-Rβ clustering, the fluorescent signal of apical images of single cells was quantified as fluorescent signal per area using the ZEN software Blue edition 3.0.

### 4.5. Protein Preparation and Western Blot Analysis 

Cells were lysed in RIPA buffer containing protease and phosphatase inhibitors (Complete; Merck, Darmstadt, Germany). Up to 15 µg of the total protein per sample was loaded onto an 8% SDS-PAGE. After electrophoresis, proteins were transferred onto a PVDF membrane (Roche, Mannheim, Germany) via semidry blotting. After blocking with 5% skim milk powder in 1× PBST, membranes were incubated with 1:1000 rabbit α-phospho-Akt (Ser473, Cell Signaling Technology, Cambridge, UK), 1:1000 rabbit α-phospho-ERK1/2 (Thr202/Tyr204, Cell Signaling Technology, Cambridge, UK), 1:1000 mouse α-PDGF-Rβ (Bio-Techne, Minneapolis, MN, USA) or 1:1000 rabbit α-β-catenin antibodies (Cell Signaling Technology, Cambridge, UK) in 1× PBS with 0.3% skim milk powder and 0.1% Tween 20 at 4 °C overnight. Following 3 washes with 1× PBS for 10 min each, the membranes were hybridized with 1:1000 alkaline phosphatase (AP)-conjugated goat α-rabbit or AP-conjugated donkey α-mouse antibodies (both from ImmunoReagents, Raleigh, NC, USA) in 1× PBS with 0.3% skim milk powder and 0.1% Tween 20. 

As loading control, blots were incubated with 1:5000 mouse α-GAP-DH antibodies (Merck, Darmstadt, Germany) in 1× PBS with 0.3% skim milk powder and 0.1% Tween 20 overnight, followed by additional hybridization with 1:1000 AP-conjugated donkey α-mouse antibodies (ImmunoReagents, Raleigh, NC, USA) in 1× PBS with 0.3% skim milk powder and 0.1% Tween 20 at room temperature for 1 h. 

For visualization, membranes were washed twice with maleic acid buffer (0.1 M maleic acid, 0.15 M NaCl, pH 7.5) for 15 min and once with detection buffer (0.1 M Tris-HCl, 0.1 M NaCl, pH 9.5) for 5 min. Following incubation with CDP-Star substrate for 5 min, in accordance with the manufacturer’s recommendations (CDP-Star, Thermo Fisher Scientific, Waltham, MA, USA), membranes were documented with an iBrightCL1000 Imaging System (Thermo Fisher Scientific, Waltham, MA, USA).

### 4.6. Statistical Analysis

All calculations and statistical analyses were performed using EXCEL for MAC version 16.85 (Microsoft, Redmond, WA, USA) and SPSS version 29.0 (IBM, Armonk, NY, USA). For data presentation, the mean and standard error of the mean (SEM) were calculated and shown as indicated. For comparison of two groups of mean variables, Student’s *t*-test was used, and for comparison of more than two groups, one-way ANOVA was used. For data that met the assumption of homogeneity of variances, a least significant difference (LSD) post hoc test was employed, and for data not meeting the criteria, a Games-Howell post hoc test was performed. *p*-values of less than 0.05 were considered statistically significant. 

## Data Availability

The original contributions presented in the study are included in the article, further inquiries can be directed to the corresponding author.
